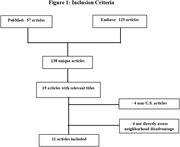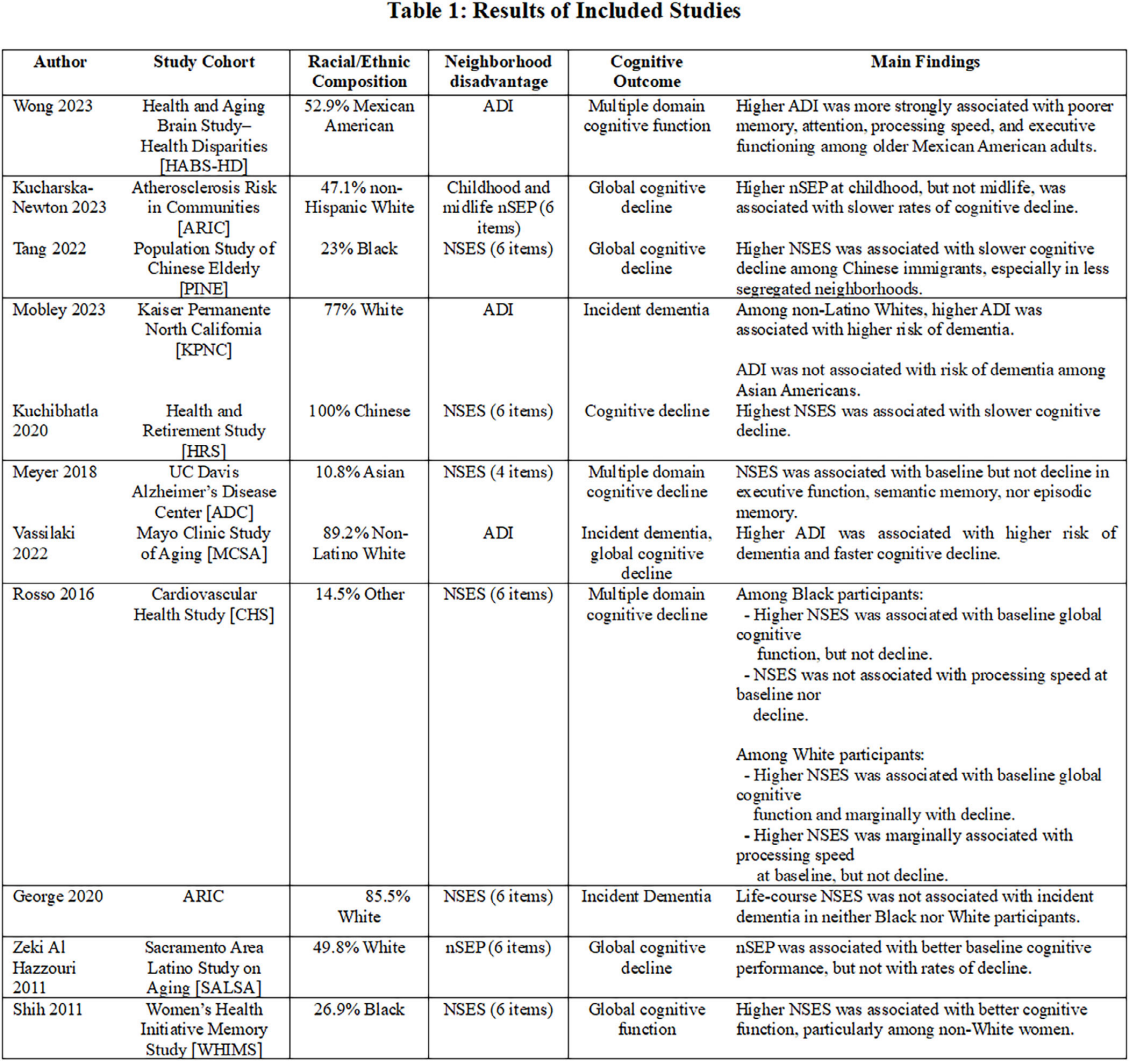# Neighborhood Disadvantage and Cognitive Outcomes: A Narrative Review

**DOI:** 10.1002/alz.087812

**Published:** 2025-01-09

**Authors:** Zeinah Al‐darsani, Erin R Kulick

**Affiliations:** ^1^ Temple University College of Public Health, Philadelphia, PA USA

## Abstract

**Background:**

The number of individuals with age‐related cognitive impairment is projected to increase at an unprecedented rate over the next few decades due to demographic shifts. Recent research endeavors have been increasingly aimed at understanding risk factors at the neighborhood level, notably socioeconomic status (SES). This review aims to provide insight into the current state of knowledge on the role of neighborhood disadvantage, defined by neighborhood SES, on late‐life cognitive outcomes.

**Method:**

Out of 138 articles meeting our criteria in PubMed and Embase, 11 U.S.‐based studies were included in this review. We focused on U.S.‐based studies because social and structural factors may vary across countries, potentially influencing the relationship between neighborhood disadvantage and cognitive outcomes.

**Result:**

Nine of the included studies were longitudinal and 2 were cross‐sectional. Three articles used the Area Deprivation Index (ADI), while 8 used a 4 or 6 item US‐Census‐based measure of neighborhood socioeconomic status (NSES) or position (nSEP) to assess neighborhood disadvantage. Three articles reported incident dementia, 5 on global cognitive decline, 2 on domain‐specific cognitive decline, 1 on domain‐specific cognitive function cross‐sectionally, and 1 on global cognition cross‐sectionally. Overall, 73% (n = 8) studies showed a significant association between neighborhood disadvantage and cognitive outcomes. Neighborhood disadvantage was not more strongly associated with a particular cognitive outcome, and results did not seem to differ based on the method of assessing neighborhood disadvantage. Most of the included studies comprised ethnically diverse participants, enhancing the generalizability of the results to the broader U.S. population and allowing exploration of ethnoracial‐specific associations. Among the 8 diverse included studies, 4 reported ethnoracial differences on the association between neighborhood disadvantage and cognitive outcomes. One study found that childhood nSEP, rather than midlife NSEP, was associated with cognitive decline.

**Conclusion:**

Neighborhood disadvantage may be associated with poorer cognition. However, additional research is needed to unequivocally establish an association between neighborhood disadvantage and cognitive outcomes due to the limited extant literature. Nevertheless, this review provides insights for future research, including investigating the influence of other structural health determinants on the relationship between neighborhood disadvantage and late‐life cognition, and as well as applying life‐course approaches to assessments of neighborhood disadvantage.